# Structural Characterization of the Avidin Interactions with Fluorescent Pyrene-Conjugates: 1-Biotinylpyrene and 1-Desthiobiotinylpyrene ^†^

**DOI:** 10.3390/molecules21101270

**Published:** 2016-09-22

**Authors:** Paweł Strzelczyk, Damian Plażuk, Janusz Zakrzewski, Grzegorz Bujacz

**Affiliations:** 1 Institute of Technical Biochemistry, Faculty of Biotechnology and Food Sciences, Lodz University of Technology, 90-924 Łódź, Stefanowskiego 4/10, Poland; pavol.strzelczyk@gmail.com; 2 Department of Organic Chemistry, Faculty of Chemistry, University of Lodz, 91-403 Łódź, Tamka 12, Poland; damplaz@uni.lodz.pl (D.P.); janzak@uni.lodz.pl (J.Z.)

**Keywords:** crystal structure, avidin, pyrene, biotin, 1-biotinylpyrene, 1-desthiobiotinylpyrene, protein crystallography, protein-ligand interaction

## Abstract

Avidin is a tetrameric protein that belongs to the calycin superfamily. It has been studied mainly because of its extraordinary affinity to biotin, which led to a wide range of applications based on the avidin-biotin system. In the present study, we report the first crystal structures of avidin in a complex with two novel fluorescent pyrene derivatives: 1-biotinylpyrene (B9P) and 1-desthiobiotinylpyrene (D9P). The crystal structures were solved by molecular replacement using the coordinates of avidin molecule as a starting model and the final models of avidin/B9P and avidin/D9P were refined to resolutions of 2.0 Å and 2.1 Å, respectively. Our data reveal changes in loop conformation as well as in overall fold and quaternary arrangement of the avidin upon the binding of these fluorescent probes. Moreover, the crystal structures allowed analysis of the details of the interactions between the protein and the pyrene derivatives. Structural description of the complexes will contribute to the design of conjugates for expanding the capabilities of avidin–biotin technology.

## 1. Introduction

**Figure 1:**
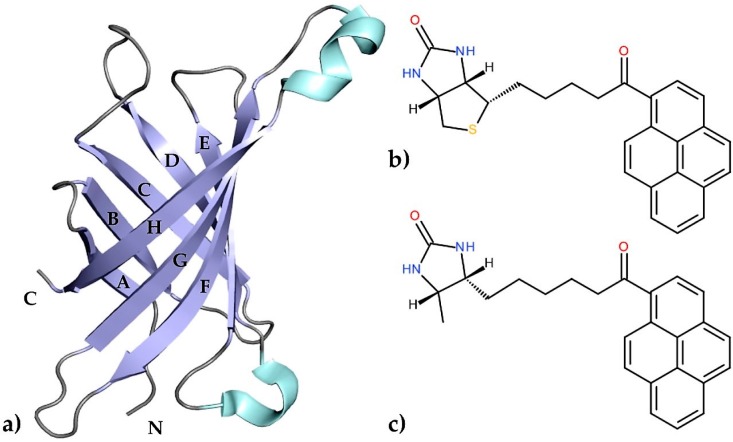
(**a**) An illustrated representation of the topology of avidin. The central eight-stranded antiparallel β-sheet, α-helixes, and loops are colored blue, cyan, and grey, respectively; (**b**) A schematic diagram of 1-biotinylpyrene (B9P) and (**c**) 1-desthiobiotinylpyrene (D9P).

Egg-white avidin, similarly to streptavidin from the bacterium *Streptomyces avidinii*, is a small, tetrameric protein which belongs to the calycin superfamily [1]. The interaction between avidin and biotin, its natural ligand, is one of the strongest non-covalent interactions known in biology (dissociation constant of ~10^−15^ M) and has been extensively characterized for more than three decades [2,3,4]. Researchers have focused on this interaction because of its extraordinarily high affinity, which became useful in many applications including immunoassays [5,6,7], biomolecule recognition [8,9,10], drug delivery [11,12,13], and cancer cell diagnosis [14,15]. This high biotin-binding affinity is based on a large number of hydrogen bonds and hydrophobic interactions due to the architecture of the binding pocket. The structural motif of this protein consists of an eight-stranded antiparallel β-barrel surrounded by flexible loops. The β-strands that form the barrel are named A–H, whereas the loops surrounding the entrance to the binding pocket are designated AB, CD, EF, and GH (Figure 1a). A previous crystallographic study confirmed that the loop CD is very flexible and can play an important role in controlling the accessibility of different ligands to the binding site. A number of biotin derivatives based on amino coupling or formation of hydrazones (C–N bond forming reactions) have been synthesized [16]. Another way to obtain vitamin H conjugates involves C–C bond formation; however, this approach requires prefunctionalized substrates [17,18].

In the present work, we have determined the first crystal structures of the avidin complexes with two fluorescent pyrene-biotin conjugates: 1-biotinylpyrene (B9P) and 1-desthiobiotinylpyrene (D9P), which were obtained via a Friedel–Crafts acylation of pyrene with biotin or desthiobiotin, respectively (Figure 1b,c). Recent studies have shown that this acylation is one of the effective C–C bond formation reactions between biotin and an aromatic system such as pyrene; moreover, such conjugates display high affinity to avidin [19]. Our structural studies reveal that the aromatic biotinylated ketones formed in this reaction may find applications in various biochemical procedures based on the biotin–avidin system, especially as fluorescent probes to visualize biochemical processes.

## 2. Results and Discussion

### 2.1. Crystallization and the Overall Features of Avidin in Its Complexes

Initial co-crystallization trials in search for diffraction quality crystals of avidin in complex with B9P failed to yield single crystals. Thus, the streak seeding method [20] was used to obtain single crystals of the avidin/B9P complex. Interestingly, initial screens for the second complex (avidin/D9P) produced crystals in several crystallization conditions.

The crystals belong to the orthorhombic space group *P*2_1_2_1_2_1_, with the unit-cell parameters *a* = 57.16, *b* = 81.95, and *c* = 107.89 for avidin/B9P and *a* = 58.97, *b* = 81.52, and *c* = 107.78 for avidin/D9P. A molecular replacement procedure found four independent avidin monomers in the asymmetric unit. The final models of the avidin/B9P and avidin/D9P complexes were refined to resolutions of 2.0 Å and 2.1 Å, respectively. The crystal structure of the avidin/B9P complex is isomorphous with the avidin/D9P complex, with the root-mean-square (r.m.s.) deviations of 0.5655 Å based on superposition of 120 C^α^ atoms from monomer C. Superposition of the C^α^ atoms of the chain A from the avidin/B9P crystal structure (PDB code 5IRU) with the ligand-free protein (PDB code 1AVE, chain A [21]) and the avidin/biotin complex (PDB code 2AVI, chain A [22]) are characterized by the r.m.s. deviations of 0.5838 Å and 0.3939 Å, respectively. These r.m.s.d. values indicate the presence of conformational changes in the main chain upon the binding of the pyrene derivative in the region of loops CD and FG.

Superposition of the C^α^ atoms of the chain C from the avidin/B9P crystal structure with the chain A from the avidin/biotin crystal structure confirms that the interactions seen in the avidin/biotin complex (PDB code 2AVI) are slightly different from those observed in the complex of avidin with B9P. This adjustment involves movement of four amino acids, Ala39-Asn42, from the top of the loop CD and six amino acids, Ile85-Lys90, from the loop FG. The largest shift is observed for C^α^ of Thr40 and Ser41 from loop CD—2.5 Å and 1.7 Å, respectively. In the case of loop FG, the largest shift is observed for C^α^ of Arg87, Asn88, and Gly89—5.8 Å, 5.4 Å, and 3.5 Å, respectively. The rest of the residues in avidin have practically the same conformation.

**Figure 2:**
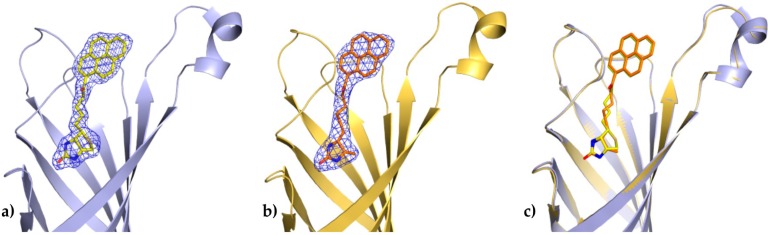
Binding site of avidin in molecule C. The coordinates of 1-biotinylpyrene (**a**) and 1-desthiobiotinylpyrene (**b**) superimposed on the final 2F_o_-F_c_ electron density maps contoured at the 1σ level; (**c**) Superposition of two avidin structures determined in this work.

In both determined structures of the avidin complexes, the calculated electron-density maps were clear and allowed tracing of almost all residues of the protein. Chain C from the avidin/B9P complex, which has a relatively similar topology to chain C from the avidin/D9P (PDB code 5IRW), was selected for structural descriptions of protein molecule. The pyrene derivative in this monomer was exceptionally well defined in difference electron-density maps and could be modeled without any ambiguity (Figure 2a).

The overall fold of avidin is characteristic for a biotin-binding protein, consisting of an eight-stranded antiparallel β-barrel. The β-strands are connected by flexible loops. A high mobility of the loops that surround the β-barrel is reflected by the observed B-factor distribution in the protein chain. B-factor values increase from the center of the β-barrel to its ends and are highest in the loop regions.

### 2.2. Fluorescence Properties

**Figure 3:**
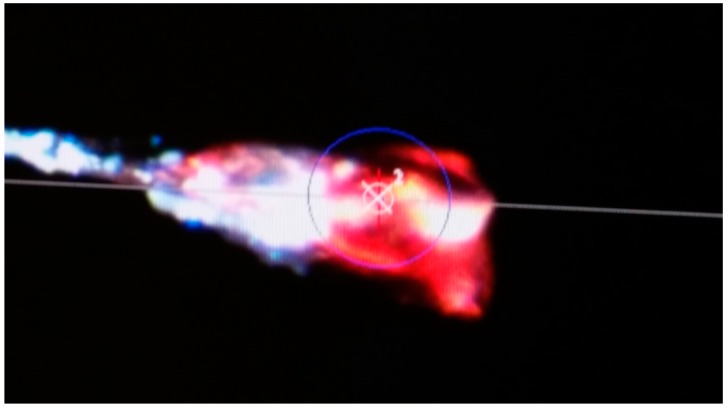
Snapshot of the avidin/B9P crystal made during data collection.

The fluorescence properties of B9P in its free form and upon binding to avidin were investigated and described earlier [19]. Shortly, free B9P excited at 355 nm displayed fluorescence emission in aqueous solutions at λ_max_ = 461 nm. The fluorescence maximum was shifted to 425 nm upon the binding of B9P to avidin. Formation of the avidin/B9P complex was also evidenced by quenching of the fluorescence from the protein tryptophan residues (342 nm) and the appearance of the emission band of the avidin/B9P at 430 nm as a result of a Förster resonance energy transfer (FRET) phenomenon [19]. Interestingly, we found that the crystal of avidin/B9P complex exhibited pink fluorescence upon excitation by a strong synchrotron X-ray beam during diffraction data collection (Figure 3).

### 2.3. N-Linked Glycosylation Site

Avidin has a single N-linked glycosylation site at Asn17. The electron density for the NAG moiety is well defined with quality similar to the density of the side chain of Asn17 for both determined crystal structures. Superposition of the avidin/B9P structure with unliganded avidin monomers (PDB code 1AVE) revealed that the conformation of the residues surrounding the Asn17 glycosylation site is highly conserved, but positions of the NAG molecules do not align with each other. In the tetragonal crystal form of free avidin, only one *N*-acetyl-d-glucosamine molecule is observed, as no electron density for any additional sugar moieties has been detected. Pugliese et al. demonstrated that the NAG moiety protrudes towards the solvent region and exhibits, in molecule B, hydrogen bonding interaction to the main-chain atom of Gly15 (PDB code 1AVE). The structures of avidin/B9P and avidin/D9P complexes show that the NAG moiety forms interactions with the backbone atoms of Gly15 in monomer D and C, respectively. In addition, one of the hydroxyl groups of NAG in monomer B interacts with the side-chain of Lys9 from the strand A. In these structures, the NAG molecule rotates straight out of the protein towards the solvent region. The next sugar moieties were in fragmented density in an F_o_-F_c_ map; thus, after refinement disappeared, only one sugar from the glycosylated chain was left in the final model.

### 2.4. Crystal Packing and Intermolecular Interactions

The final models of the avidin complexes with pyrene derivatives contain four molecules (A–D) in the asymmetric unit and analysis of the protein interfaces between subunits using the *PDBePISA* server shows that avidin exists as a tetramer in solution, which correlates with the gel-filtration chromatographic profile. These subunits show polar and apolar intermolecular interactions. The tetrameric assembly may be considered as a dimer of dimers [22]. The total contact surface area of the A–B and C–D dimers interface (PDB code 5IRU) is approximately 1766 Å^2^ and 1751 Å^2^, respectively (Table 1), as calculated by *PISA* [23,24]. The interaction interface between these subunits is formed mainly by amino acid residues in stands E–G and loop EF. The interaction between monomers A–C and B–D (a total buried surface area of 622 Å^2^ and 634 Å^2^, respectively) includes only three residues that form in total six hydrogen bonds. The main-chain carbonyl O atoms of Ala39 and Lys111 form hydrogen bonds to the side-chain amides of Lys111 and the nitrogen atom NE of Arg114, respectively. Additionally, the main-chain carbonyl of Thr113 is within a hydrogen-bonding distance to the amide of Val115. These amino acids are generally localized in β-strand H. The loop GH includes Trp110, which plays an important role in stabilizing the fluorescent ligand in the binding pocket and preserves the integrity of the tetramer. The smallest intermolecular contact surface (147 Å^2^) is found between molecules A and D, where neither hydrophobic contacts nor hydrogen bonds are present (Table 1). This result shows that the interaction between pairs of dimers A–B and C–D is much stronger than the interaction between these dimers.

### 2.5. Binding of the Pyrene Derivatives

**Figure 4:**
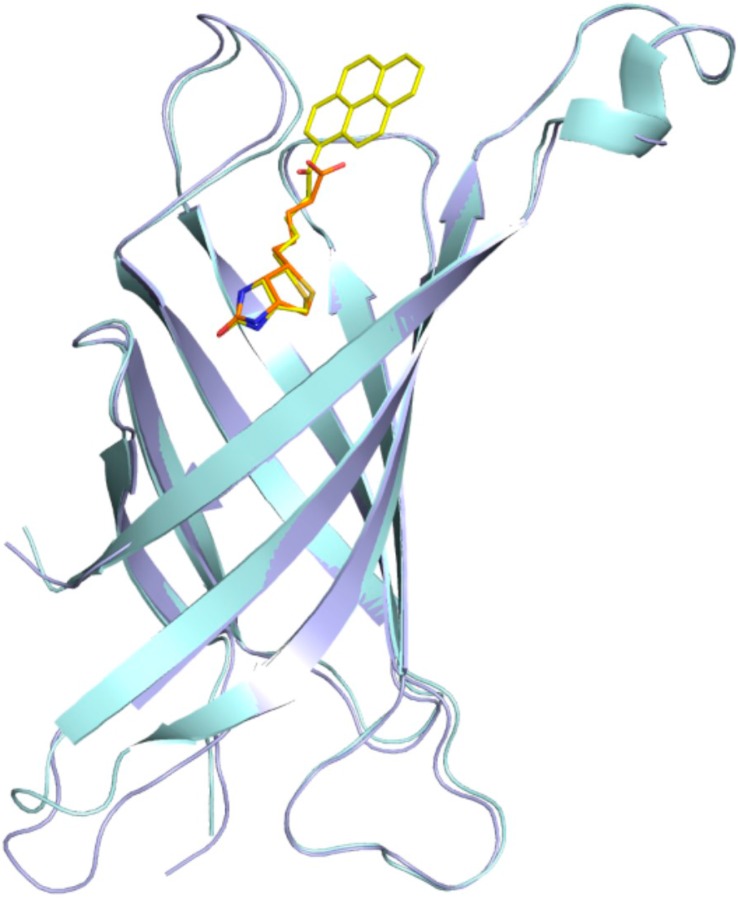
Superposition of the avidin–biotin structure (cyan/orange) with the avidin/B9P structure (light blue/yellow). The structures were superimposed on corresponding C^α^ atoms.

The binding cavity in each subunit of avidin is occupied by a single ligand molecule with the same orientation, with the ring system of biotin and desthiobiotin moieties hidden deeply inside the β-barrel, while the pyrene moiety is located at the barrel entrance. Superposition of the crystal structures described here with an avidin–biotin complex (PDB code 2AVI) shows that certain canonical interactions in the hydrophobic binding pocket and the position of the ligands remain the same (Figure 4). In both avidin–pyrene conjugate complexes, well-defined electron density allowed for the determination of the position of B9P and D9P (Figure 2) in monomer C, but the pyrene moieties of D9P are not equally well visible in the electron density maps in all monomers that form the avidin tetramer. Photo-degradation of the pyrene ring is most likely observed in two monomers in the avidin/D9P complex, and only part of the ligand was included in the final model. This degradation is more effective for the monomer where the β-barrel opening site is exposed to the solvent channels. For molecules involved in more extensive crystal contacts, less degradation was observed. Another indication of photo-degradation was pink fluorescence during diffraction data collection experiments of the crystals of both complexes (Figure 3).

Chain C was selected for structural description of the interactions between the protein and pyrene conjugates. The pyrene derivatives bound in an elongated manner in the β-barrel and filled almost all available space of the binding pocket. These ligand molecules match the geometry of the deepest part of the binding site very well. The biotin/desthiobiotin moiety reaches the bottom of the barrel where a few polar side chains allow for the creation of hydrogen bonds to the suitable heteroatom partners of the ligand molecules. The B9P molecule consists of the ureido ring, tetrahydrothiophene ring, valeric acid, and the pyrene moiety, while D9P molecule includes the methyl group and methylene bridge instead of the tetrahydrothiophene ring. In this study, both conjugates were used as ligands during the crystallization experiments. The conformation of the B9P or D9P molecules in the crystal structures of their complexes is almost identical.

**Figure 5:**
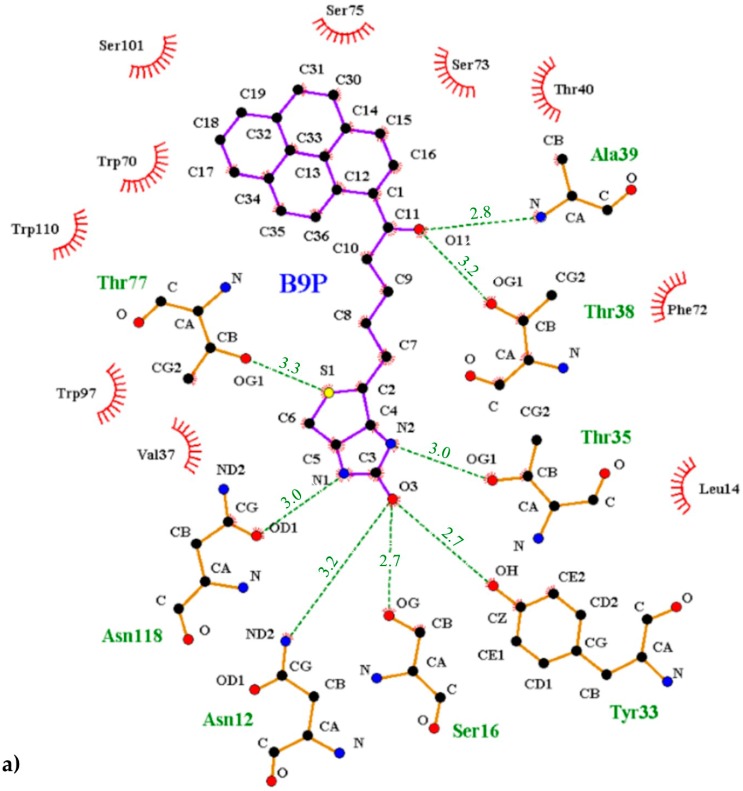

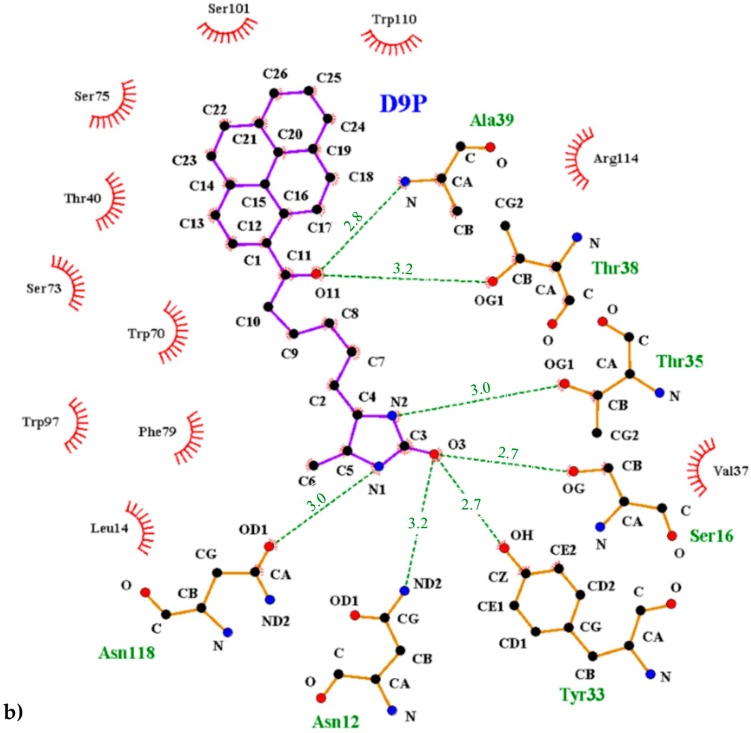
Interactions between the avidin and pyrene conjugates: (**a**) 1-biotinylpyrene and (**b**) 1-desthiobiotinylpyrene. The scheme was prepared with the LIGPLOT+ program [25]. Hydrogen bonds are indicated with a green dashed line, and the residues involved in hydrophobic interactions are shown with red rays.

The interior of the binding pocket is coated by hydrophobic side chains of eight β-strands (named from A to H) of the β-barrel and by flexible loops connecting them. Although the B9P/D9P ligand is docked in a predominantly hydrophobic cavity (formed by several aromatic residues, including Trp70, Phe72, Phe79, and Trp97, as well as Trp110 from a symmetry-related molecule), hydrogen bonds are mainly responsible for its orientation in the binding pocket. The carbonyl group of the ureido ring of the B9P/D9P molecule is anchored by the side chains of Asn12 and Ser16 from loop AB and Tyr33 from strand C. The amino group of the ureido fragment is coordinated by the side-chain hydroxyl groups of Asn118 and Thr35 from strand H and loop CD, respectively. The sulfur atom from the tetrahydrothiophene ring of B9P acts as an acceptor for the hydroxyl group of Thr77, while in the case of D9P there is no equivalent interaction. The aliphatic valeryl fragment of B9P/D9P is located in the middle part of the cavity and the carbonyl group from this part of the ligand forms a pair of hydrogen bonds with the nitrogen atom of Ala39 and the hydroxyl group of Thr38, localized in the most flexible loop CD. The pyrene moiety, which consists of four electron-rich rings, is located at the entrance of the binding pocket, where it is surrounded by polar amino acids (Thr40, Asn42, Ser73, Ser75, Ser101, and Arg114) in loops CD, EF, and GH. The side chains of these residues play an important role in stabilizing the ligands (Figure 5). The hypsochromic shift of the fluorescence maximum upon avidin/B9P complex creation [19] may indicate that the avidin binding loops in the area of pyrene are less polar than the bulk solvent.

## 3. Materials and Methods

### 3.1. Materials

**Figure 6:**
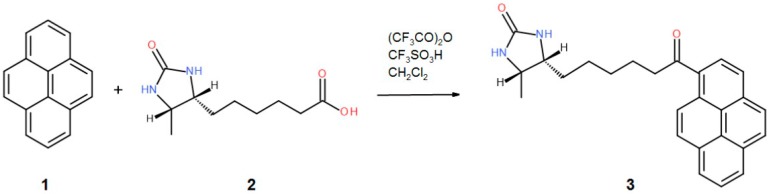
A schematic diagram depicting the synthesis of the 1-desthiobiotinylpyrene (D9P).

Avidin from hen egg white (lyophilized powder) was purchased from Lee Biosolutions, Inc. (Maryland Heights, MO, USA). Crystallization components and plates were obtained from Hampton Research (Aliso Viejo, CA, USA). Crystallization screens were purchased from Hampton Research (Aliso Viejo, CA, USA) and Molecular Dimensions (Newmarket, UK). The 1-desthiobiotinylpyrene (D9P) was obtained by chemical synthesis based on the Friedel-Crafts acylation (Figure 6), while the 1-biotinylpyrene (B9P) was synthesized according to a published procedure [19].

### 3.2. Methods

#### 3.2.1. General Procedure of the Synthesis of 1-Desthiobiotinylpyrene (D9P)

An amount of 227 mg (150 µL, 1.08 mmol) of the trifluoroacetic anhydride was added to a solution of 214 mg (1.0 mmol) of d-desthiobiotin **2**. After 1 min of stirring at RT, 202 mg (1.0 mmol) of pyrene **1** was added, followed by an addition of 88 µL (1.0 mmol) of trifluoromethanesulfonic acid. After 2 h of stirring at RT, the reaction mixture was quenched by an addition of 10 mL of sodium bicarbonate, and the product was extracted with dichloromethane. The organic layer was washed with brine, dried over magnesium sulfate, and evaporated. Chromatography on silica gel using DCM-Methanol 95-5 gave pure **3** in a 41% yield (163 mg)—Figure 6.

^1^H-NMR (CDCl_3_, 600.26 MHz) δ 8.87 (d, *J* = 9.3 Hz, 1H), 8.31 (d, *J* = 8.0 Hz, 1H), 8.25 (d, *J* = 7.6 Hz, 1H), 8.24 (d, *J* = 7.6 Hz, 1H), 8.21 (t, *J* = 9.4 Hz, 1H), 8.18 (d, *J* = 7.5 Hz, 1H), 8.16 (d, *J* = 8.3 Hz, 1H), 8.08 (d, *J* = 9.1 Hz, 1H), 8.05 (d, *J* = 7.6 Hz, 1H), 3.86–3.81 (m, 1H), 3.72–3.69 (m, 1H), 3.22 (t, *J* = 7.3 Hz, 2H), 1.90–1.85 (m, 2H), 1.57–1.27 (m, 6H), 1.12 (d, *J* = 6.5 Hz, 3H); ^13^C-NMR (CDCl_3_, 150.94 MHz) δ 204.9, 163.2, 33.7, 132.7, 131.1, 130.6, 129.6, 129.5, 129.3, 127.1, 126.4, 126.3, 126.0 (two carbons), 125.1, 124.7, 124.4, 124.0, 56.1, 51.5, 42.4, 29.5, 29.2, 26.4, 24.7, 15.7; Elemental analysis calc. for C_26_H_26_N_2_O_2_.1/2CH_3_OH C-76.78, H-6.81, N-8.03 found C-76.83, H-6.95, N-7.51.

#### 3.2.2. Complex Formation and Crystallization

Commercially obtained avidin was further purified by gel-filtration chromatography on an ÄKTA FPLC system (Amersham Biosciences, Uppsala, Sweden). The purified protein was concentrated on Vivaspin filters with a 10-kDa cutoff (Sartorius, Goettingen, Germany) on a centrifuge under 7000 rcf (Eppendorf, Hamburg, Germany) and was used for complex formation. The complexes of avidin/B9P and avidin/D9P were obtained by a co-crystallization method. The 1-mM solution was mixed with the 10-fold access of ligands to form complexes. The mixtures were incubated for 6 h. Initial screening for crystallization conditions was carried out with commercially available screens: Index Screen (Hampton Research), PEG/Ion Screen (Hampton Research), and Morpheus (Molecular Dimensions). Crystallization was performed manually in 24-well plates and carried out in hanging drops using a vapor diffusion method at 293 K. The streak seeding method was used to obtain single crystals of the avidin/B9P complex. In this technique, horse tail hair was used as a seeding wand, which was placed into contact with the surface of the parent crystal and then drawn through the new drop. We noted that the best results were observed in the drops at pH 6.5. The crystallization reservoir for avidin/B9P was composed of 28% *w*/*v* polyethylene glycol monomethyl ether 2000, 0.1 M of Bis-Tris, 0.1 M of trimethylamine hydrochloride, and the drops were composed of 1 μL of protein–ligand solution and 1 μL of reservoir solution. In the case of avidin/D9P, crystals were grown using a reservoir solution consisting of 100 mM of imidazole/MES monohydrate (acid) buffer pH 6.5, 10% *w*/*v* polyethylene glycol 20,000, 20% PEG 500 MME, 30 mM of magnesium chloride hexahydrate, and 30 mM of calcium chloride dehydrate. The drops were composed of 1 μL of protein–ligand solution and 0.5 μL of reservoir solution. The crystals of the complexes appeared after two weeks (avidin/B9P) and one week (avidin/D9P).

#### 3.2.3. X-ray Data Collection and Processing

X-ray diffraction data from crystals of each complex were collected using the P13 beamline on the PETRA III ring of the DESY synchrotron (Hamburg, Germany). The crystals were flash-cooled directly at 100 K in a cold nitrogen-gas stream without additional cryoprotection. Diffraction data were indexed, integrated, and scaled using *XDS* [26]. Table 2 gives the statistics of data collection and processing.

#### 3.2.4. Structure Determination and Refinement

The structure of the complexes was solved by molecular replacement using *MOLREP* [27] with a single monomer of avidin (PDB code 3VGW [28] or 4I60 [29]) used as a search model. Manual model rebuilding according to electron-density maps was performed for both structures in *Coot* [30,31]. Maximum-likelihood refinement of both structures was carried out in *REFMAC5* [32] from the *CCP4* suite [33]. In both structures, the presence of the ligands was clearly seen in the different electron-density maps generated with phases calculated from only the protein model. Geometrical restraints for both ligands were generated in *Sketcher* from the *CCP4* package [33]. The refinement statistics are summarized in Table 2.

## 4. Conclusions

In the present study, we determined the first crystal structures of hen egg white avidin in a complex with highly potent fluorescent pyrene conjugates, 1-biotinylpyrene (B9P) and 1-desthiobiotinylpyrene (D9P), at resolutions of 2.0 Å and 2.1 Å, respectively. Pyrene derivatives were obtained via a Friedel–Crafts acylation, which is an effective C–C bond formation reaction. These two structures provide information on the overall architecture of avidin and detail the avidin–ligand interactions in the binding cavity. Although the B9P/D9P ligand is docked in a predominantly hydrophobic cavity, hydrogen bonds are mainly responsible for its orientation in the binding pocket. Our structural studies provide valuable data for the characterization of avidin and reveal that the aromatic biotinylated ketones (B9P and D9P) may find application in various biochemical procedures based on the biotin–avidin system.

## Figures and Tables

**Table 1:** Statistics of intermolecular interactions in the asymmetric unit of the avidin/B9P complex.

Chains	No. of Interface Residues	Interface Area (Å^2^)	No. of Hydrogen Bonds	No. of Potential Salt Bridges
A:B	49	1766	32	7
A:C	15	622	6	0
A:D	4	147	0	0
C:D	48	1751	33	9
B:D	16	634	6	0
B:C	4	148	0	0

**Table 2:** X-ray data collection and crystal structure refinement statistics. Values in parentheses are for the last resolution shell.

PDB Code	5IRU	5IRW
**X-ray Data Collection**		
Radiation source	PETRA III, EMBL C/O DESY	
Wavelength (Å)	0.99999	0.99999
Temperature (K)	100	100
Space group	*P*2_1_2_1_2_1_	*P*2_1_2_1_2_1_
Unit cell parameters (Å)	*a* = 57.16, *b* = 81.95, *c* = 107.89	*a* = 58.97, *b* = 81.52, *c* = 107.78
Solvent content (%)	44.1	45.5
Resolution range (Å)	46.88–2.00 (2.05–2.00)	43.68–2.10 (2.15–2.10)
Completeness (%)	99.4 (99.4)	99.9 (99.9)
<I/σ(I)>	12.48 (3.61)	24.34 (3.35)
Total reflections	222,713 (30,904)	202,435 (26,978)
Unique reflections	34,767 (4669)	30,993 (3971)
Redundancy	6.41 (6.62)	6.53 (6.79)
R_int_ (%)	8.20 (50.80) ^†^	4.10 (69.50) ^†^
CC1/2	99.6 (95.8)	100.0 (92.7)
**Refinement**		
R_work_	0.222 ^‡^	0.195 ^‡^
R_free_	0.265 ^‡^	0.231 ^‡^
No. of atoms (non-H):		
Protein	3828	3791
H_2_O	227	112
B9P	124	-
D9P	-	95
R.m.s. deviations from ideal:		
Bond lengths (Å)	0.02	0.02
Bond angles (°)	1.98	1.96
Average B factor (Å^2^)	53.58	67.38
Ramachandran plot statistics:		
Favoured regions (%)	99.0	97.0
Allowed regions (%)	1.0	3.0

^†^ $R_{\mathrm{int}}=\sum_{hkl}\sum_{i}|I_{i}\left(hkl\right)-\langle I\left(hkl\right)\rangle |/\sum_{hkl}\sum_{i}I_{i}\left(hkl\right)$, where $I_{i}\left(hkl\right)$ is the intensity of the *i*-th observation of reflection *hkl*; ^‡^ $R=\sum_{hkl}\left|\left|F_{obs}\right|-F_{calc}\right|/\sum_{hkl}\left|F_{obs}\right|$ for all reflections, where $F_{obs}$ and $F_{calc}$ are the observed and calculated structure factors, respectively. $R_{free}$ is calculated in the same manner for the test reflections, which were randomly selected and excluded from the refinement.
